# Supplementary material for: Survival features, prognostic factors, and determinants of diagnosis and treatment among Iranian patients with pancreatic cancer, a prospective study
Source: PLoS One. 2020 Dec 4;15(12):e0243511. doi: 10.1371/journal.pone.0243511 (PMC7717574; doi:10.1371/journal.pone.0243511)
Supplement: S1 File — (DOCX) [file pone.0243511.s001.docx]

**S1 File**

**Definition of opioids, opiates, and different opium types**

Opiates are a subgroup of opioids containing the various products derived from the opium poppy plant, including opium, morphine and heroin. Opioids is a generic term that refers both to opiates and their synthetic analogues (mainly pharmaceutical opioids) and compounds synthesized in the body (1).

The main opiate types used in the region are raw opium (teriak), refined opium (shireh or opium juice), opium dross (sukhteh), and heroin. Raw opium is the air-dried extract of the opium poppy plant that is acquired through ripening the poppy capsules. Raw opium can be ingested or smoked after direct heating with special devices (2). Opium dross is the remnants of smoked opium and can only be ingested. Refined opium is obtained from boiling the opium dross (with or without adding raw opium) in water and filtering the mixture a few times and then evaporating the filtrate (2). Refined opium can be ingested or smoked by indirect heating using special devices. Heroin is typically injected into a vein, but also can be smoked.

**References**

1. United Nations Office on Drugs and Crime. World Drug Report 2019 [Internet]. 2019. Available from: https://wdr.unodc.org/wdr2019/

2. Khademi H, Malekzadeh R, Pourshams A, Jafari E, Salahi R, Semnani S, et al. Opium use and mortality in Golestan Cohort Study: prospective cohort study of 50 000 adults in Iran. BMJ. 2012 Apr 17;344:e2502.
