# Supplementary material for: Survival features, prognostic factors, and determinants of diagnosis and treatment among Iranian patients with pancreatic cancer, a prospective study
Source: PLoS One. 2020 Dec 4;15(12):e0243511. doi: 10.1371/journal.pone.0243511 (PMC7717574; doi:10.1371/journal.pone.0243511)
Supplement: S2 File — (DOCX) [file pone.0243511.s002.docx]

**S2 File**

We used the TNM classification method by the American Joint Committee on Cancer (AJCC) as presented in the table below to identify the stage of pancreatic cancer

**Tumor**

T-x: Primary tumor cannot be assessed

T-0: No evidence of primary tumor

T-is: Carcinoma in situ

T-1: Tumor limited to the pancreas, < 2 cm

T-2: Tumor limited to the pancreas, > 2 cm

T-3: Tumor extension beyond the pancreas (duodenum, bile duct, portal or superior mesenteric vein)

T-4: Tumor involving the celiac axis and superior mesenteric arteries

**Regional lymph nodes**

N-x: Regional lymph modes cannot be assessed

N-0: No regional lymph node metastasis

N-1: Regional lymph node metastasis

**Distant metastasis**

M-x: Distant metastasis cannot be assessed

M-0: No distant metastasis

M-1: Distant metastasis

**Staging**

Stage 0: Tis, N0, M0

Stage IA: T1, N0, M0

Stage IB: T2, N0, M0

Stage IIA: T3, N0, M0

Stage IIB: T1, N1, M0 or T2, N1, M0, or T3, N1, M0

Stage III: T4, any N, M0

Stage IV: any T, any M, M1
