# Supplementary material for: Survival features, prognostic factors, and determinants of diagnosis and treatment among Iranian patients with pancreatic cancer, a prospective study
Source: PLoS One. 2020 Dec 4;15(12):e0243511. doi: 10.1371/journal.pone.0243511 (PMC7717574; doi:10.1371/journal.pone.0243511)
Supplement: S1 Table — (DOCX) [file pone.0243511.s003.docx]

**S1 Table.** Estimates of overall survival rates by clinical symptoms upon diagnosis among pancreatic cancer patients

| **Characteristics** | **No (%)** | **Median Survival (month)** | **P-value ¶** | **1 year**  **Survival %** | **3 years survival %** | **5 years survival %** |
| --- | --- | --- | --- | --- | --- | --- |
| **Abdominal pain** | | | | | | |
| No | 81 (17.5) | 12.1 | **<0.001** | 46.9 | 11.1 | 4.9 |
| Yes | 380 (82.4) | 6.0 |  | 21.8 | 2.8 | 0.7 |
| **Unintentional weight loss** | | | | | | |
| No | 87 (18.8) | 9.8 | **0.002** | 39.0 | 6.9 | 2.3 |
| Yes | 374 (81.3) | 6.1 |  | 23.2 | 3.7 | 1.3 |
| **Dark-colored urine** | | | | | | |
| No | 204 (44.2) | 6.9 | 0.17 | 28.9 | 3.9 | 1.4 |
| Yes | 257 (55.7) | 6.1 |  | 24.1 | 4.6 | 1.5 |
| **Jaundice** | | | | | | |
| No | 220 (47.7) | 6.7 | 0.78 | 24.0 | 3.1 | 1.3 |
| Yes | 241 (52.2) | 6.4 |  | 28.2 | 5.3 | 1.6 |
| **Light-colored stool** | | | | | | |
| No | 272 (59.0) | 6.6 | 0.55 | 27.5 | 3.6 | 1.4 |
| Yes | 189 (41.0) | 6.5 |  | 24.3 | 5.2 | 1.5 |
| **Constipation** | | | | | | |
| No | 274 (59.4) | 6.9 | **0.020** | 29.9 | 5.4 | 2.1 |
| Yes | 187 (40.5) | 5.8 |  | 20.8 | 2.6 | 0.5 |
| **Anorexia** | | | | | | |
| No | 279 (60.5) | 6.5 | 0.052 | 29.0 | 4.3 | 2.1 |
| Yes | 182 (39.4) | 6.5 |  | 21.9 | 4.4 | 0.5 |
| **Pruritus** | | | | | | |
| No | 278 (60.3) | 6.5 | 0.57 | 24.1 | 3.2 | 1.0 |
| Yes | 182 (39.7) | 6.5 |  | 29.5 | 6.0 | 2.1 |
| **Abdominal bloating** | | | | | | |
| No | 303 (65.7) | 6.5 | 0.29 | 25.7 | 4.6 | 1.6 |
| Yes | 158 (34.2) | 6.5 |  | 27.2 | 3.8 | 1.2 |
| **Nausea** | | | | | | |
| No | 329 (71.3) | 6.9 | 0.15 | 27.6 | 3.6 | 0.6 |
| Yes | 132 (28.6) | 5.7 |  | 22.7 | 6.0 | 3.7 |
| **Fever** | | | | | | |
| No | 342 (74.1) | 6.9 | 0.32 | 26.6 | 4.3 | 1.1 |
| Yes | 119 (25.8) | 5.7 |  | 25.2 | 4.2 | 2.5 |
| **Shivering** | | | | | | |
| No | 353 (76.5) | 6.8 | 0.12 | 26.9 | 5.1 | 1.7 |
| Yes | 108 (23.4) | 5.8 |  | 24.0 | 1.8 | 0.9 |
| **New onset diabetes *** | | | | | | |
| No | 408 (88.5) | 6.4 | 0.46 | 27.2 | 9.1 | 4.6 |
| Yes | 53 (11.5) | 6.9 |  | 35.2 | 6.2 | 3.1 |
| **Steatorrhea** | | | | | | |
| No | 417 (90.4) | 6.7 | 0.57 | 26.1 | 4.3 | 1.4 |
| Yes | 44 (9.5) | 5.2 |  | 27.2 | 4.5 | 2.2 |
| **Number of symptoms** | | | | | | |
| 1 - 3 | 117 (25.3) | 8.7 | **0.013** | 39.4 | 14.6 | 6.6 |
| 4 – 5 | 105 (22.7) | 6.9 |  | 25.8 | 7.3 | 4.4 |
| 6 – 7 | 116 (25.1) | 6.1 |  | 24.3 | 8.4 | 3.8 |
| ≥ 8 | 123 (26.6) | 5.7 |  | 23.2 | 5.1 | 2.8 |

**¶** Log-rank test **;** * New onset diabetes was defined as being diagnosed with diabetes mellitus in the recent 2 years
