# Supplementary material for: Survival features, prognostic factors, and determinants of diagnosis and treatment among Iranian patients with pancreatic cancer, a prospective study
Source: PLoS One. 2020 Dec 4;15(12):e0243511. doi: 10.1371/journal.pone.0243511 (PMC7717574; doi:10.1371/journal.pone.0243511)
Supplement: S1 Questionnaire — (PDF) [file pone.0243511.s004.pdf]

PBD||__||__||__||__||__||__| |__||__| |__|

1.1 .1**نام** : .... 1.1.2**نام خانوادگی** : ....

معاینه آنتروپومتریک (وزن و قد بدون کفش و لباس‌های سنگین اندازه‌گیری شود).

1.1.3. **قد** به cm :..................... 1.1.4. **وزن** به Kg :.....................

1.2.**محل انجام مصاحبه**: |__|

1)بیمارستان شریعتی 2)مرکز Cohort گلستان 3) بیمارستان آتیه

4)کلینیک مسعود 5) بیمارستان فیروزگر 6) بیمارستان امام 7)سایر: ....

1.5. **تاریخ مصاحبه**: روز/ماه/سال ..../......./........

1.6. **محل تولد**: استان.............. شهرستان .... شهر .... روستا.................................

. مشخصات **محل سکونت فعلی** : **1- شهر** **2-روستا** ***<u>چند سال است؟</u>***

استان:.............. شهرستان:............... شهر:................. روستا:...............

خیابان:.................. کوچه:................ پلاک:.......................

تلفن ثابت منزل:.................. پیش شماره منزل :.................. تلفن همراه:................................

مشخصات همراه بیمار یا یکی از اعضای خانواده یا دوستان و همکاران :

نام : .... نام خانوادگی :................................. نسبت آشنایی:...

تلفن ثابت منزل:.................. پیش شماره منزل :.................. تلفن همراه:...............................

1.7. **جنس** : |__| 1)مرد 2)زن

1.8.**تاریخ تولد**: روز/ماه / سال ..../......./........

1.9.**سن** (سال کامل) : ....

1.10.1.**نژاد پدر بیمار**: |__|

1)فارس 2)ترک 3)ترکمن 4)گیلانی 5)مازندرانی 6)کرد 7) لر 8)سایر(نام ببرید) :..............

1.10.2.**نژاد مادربیمار** : |__|

1)فارس 2)ترک 3)ترکمن 4)گیلانی 5)مازندرانی 6)کرد 7) لر 8)سایر(نام ببرید) :...........

1.11.1 **وضعیت تاهل** : |__|

1)مجرد 2)متاهل 3)همسر مرده، بیوه 4)مطلقه 5)سایر:..................

1.11.2. **سن بیمار در زمان اولین ازدواج: |__||__|**

1.12. **سطح تحصیلات بیمار**: |__|

1) بی سواد 2)مساوی یا کمتر از 5 سال 3)بین 6 – 8 سال 4) بین 9–12 سال 5) تحصیلات عالی

1.13**سطح تحصیل سرپرست خانواده**: |__|

1)بی سواد 2) مساوی یا کمتر از 5سال 3) بین 6–8 سال 4) بین 9 تا 12 سال 5) تحصیلات عالی

1.14.**هویت فرد پاسخگو** به سوالات : |__| و |__|

1) خود بیمار 2) همسر بیمار 3) خویشاوندان دیگر 4) سایر افراد:. (نام ببرید) ....

. علایم و نشانه های بیماری فعلی

4.1. آیا شما تا به حال علایم زیر راداشته‌اید؟ اگر بلی، اولین بار کی دچار این علایم شدید؟ (**معیار طولانی ترین علامت است**)

4.2. زمان شروع علایم:|__|

1) کمتر از یک ماه قبل 2)بین 1 تا 3 ماه قبل

3) بین 3 تا 6 ماه قبل 4)بیش از 6 ماه قبل 5) هرگز

شماره	علایم	1)بلی 2) خیر
4.1.1	زردی	|__|
4.1.2	خارش	|__|
4.1.3	تب	|__|
4.1.4	لرز	|__|
4.1.5	یبوست	|__|
4.1.6	درد شکم	|__|
4.1.7	تغییر رنگ ادرار	|__|
4.1.8	تغییر رنگ مدفوع	|__|

4.3. آیا **کاهش وزن** داشته‌اید؟ |__| 1) بلی 2) خیر

4.3.1. اگر بلی ، چند کیلو گرم ؟..............(نا مشخص = 98) |__||__|

4.3.2. آیا این کاهش وزن ارادی بوده است ؟(یعنی با رژیم غذایی) |__|

1) بلی 2) خیر 8) کاهش وزن نداشته ام

5. سابقه شخصی بیماری :

شماره	بیماری	1)بلی 2)خیر<br>3) نمی دانم	مدت بیماری (سال)<br>**اگر خیر = کد 98**
5.1.3	سابقه بیماری دیابت	|__|	|__||__|
5.1.6	سابقه بیماری ایسکمیک قلبی (شامل سکته قلبی و نارسائی قلبی)	|__|	|__||__|
5.1.7	سابقه سکته مغزی	|__|	|__||__|
5.1.7	سابقه افزایش فشار خون	|__|	|__||__|
5.1.16	سابقه هیپرکلسترولمی	|__|	|__||__|
5.1.17	سابقه هیپرتری گلیسیریدمی	|__|	|__||__|
5.1.22	سابقه عمل جراحی کیسه صفرا	|__|	|__||__|

PBD||__||__||__||__||__||__| |__||__| |__|

6.1. آیا شما مبتلا به کانسری که توسط پزشک تشخیص داده شده باشد, بوده‌اید؟ |__| 1) بلی 2)خیر

6.1.1. اگر بلی چه نوعی از سرطان؟ |__||__|

6.1.2. اگر بلی،سن در زمان تشخیص؟ |__||__|

6.1.3. اگر بلی، آیا شیمی درمانی شده است؟|__| 1) بلی 2)خیر

6.1.4. اگر بلی، چند بار شیمی درمانی شده است؟|__| 1) بلی 2)خیر

6.1.3. اگر بلی، تاریخ اولین شیمی درمانی؟ |__||__| D/ |__||__| M / |__||__||__||__|

کد نوع سرطان: 1= دهان یا حلق، 2= مری، 3= معده، 4= روده بزرگ، 5= لوزالمعده، 6= کبد، 7= حنجره، 8= ریه، 9=پوست، 10= پستان، 11= رحم، 12= پروستات، 13= مثانه، 14= خون، 15= سایر، 16= کیسه صفرا, 17= مجاری صفراوی, 18= با منشا نامشخص.

6.3. **سابقه ابتلا به کانسر در اعضای فامیل**: |__| 1) بلی 2) خیر اگر بلی است، در جدول زیر فهرست نمایید:

نسبت فامیلی	کد نوع سرطان	سن تشخیص
|__||__|	|__||__|	|__||__|
|__||__|	|__||__|	|__||__|
|__||__|	|__||__|	|__||__|

کد نسبت فامیلی: (1) پدر، (2) مادر، (3) برادر تنی، (4) خواهر تنی، (5) برادر ناتنی، (6) خواهر ناتنی، (7) پسر، (8) دختر، (9) پدربزرگ یا مادربزرگ، (10) همسر، (11) سایر خویشاوندان خونی درجه دو (عمو، عمه, دایی، خاله، عمو و عمه‌زاده، خاله و دایی‌زاده)

7- سابقه مصرف دارو:

7.1. آیا تاکنون دارویی را به صورت منظم مصرف نموده‌اید؟ |__| 1) بلی 2) خیر

7.2. اگر بلی، نام، مدت و دفعات مصرف را فهرست نمایید (کلیه داروها از جمله داروهای ضدبارداری داروهای تقویتی):

	نام دارو	مصرف فعلی (1)بلی/ (2)خیر	مدت (سال)	دفعات مصرف 1) روزانه 2) هفتگی 3)ماهانه	**دوز(میلی گرم)**
7.2.1	آسپیرین	|__|	|__||__|	|__|	
7.2.2	استاتین‌ها *	|__|	|__||__|	|__|	
7.2.3	انسولین	|__|	|__||__|	|__|	
7.2.4	متفورمین	|__|	|__||__|	|__|	
7.2.5	گلی بن کلامید	|__|	|__||__|	|__|	
7.2.6		|__|	|__||__|	|__|	

* منظور از استاتین ها داروهای لووستاتین ،آترووستاتین ، سیموستاتین می باشد.

8.1 آیا بطور مرتب (حداقل یکبار درهفته به مدت شش ماه) **سیگار** کشیده‌اید؟ |__|　(1) بلی　(2) خیر

8.2. آیا در حال حاضر سیگار می‌کشید؟ |__|　(1) بلی　(2) خیر　(3) هیچگاه سیگار مصرف نکرده‌ام

8.3. مصرف سیگار را از زمان آغاز مصرف منظم آن فهرست نمایید:

از سن	تا سن	نوع •	تعداد در روز	روز در هفته
		|__|	|__||__|	|__|
		|__|	|__||__|	|__|
		|__|	|__||__|	|__|
		|__|	|__||__|	|__|
		|__|	|__||__|	|__|

• **نوع:** 1- سیگار فیلتردار کارخانه‌ای　2- سیگار بدون فیلتر کارخانه‌ای　3- دست‌ساز

8.4. آیا بطور مرتب (حداقل یکبار در هفته به مدت شش‌ماه) **ناس**، **قلیان**، **چپق یا پیپ** استفاده کرده‌اید؟|__|　1)بلی　2) خیر

در صورت بلی، نوع و مدت مصرف آن را مشخص نمایید:

	نوع	آیا مصرف شده؟ (1) بلی	اگر مصرف شده باشد				توضیحات
			از سن	تا سن	دفعات معمول روزانه	روز در هفته	
8.4.1	ناس	|__|	|__||__|	|__||__|	|__||__|	|__|	
8.4.2	چپق	|__|	|__||__|	|__||__|	|__||__|	|__|	
8.4.3	پیپ	|__|	|__||__|	|__||__|	|__||__|	|__|	
8.4.4	قلیان	|__|	|__||__|	|__||__|	|__||__|	|__|	

8.5. آیا بطور مرتب (حداقل یکبار در هفته در مدت شش ماه) **تریاک ـ موادمخدر** استفاده کرده‌اید؟ |__|　(1) بلی　(2) خیر

8.5.1. اگر بلی ، نوع و مدت مصرف آن را مشخص نمایید:

نوع	آیا مصرف شده؟ (1) بلی	اگر مصرف شده باشد					توضیحات
		از سن	تا سن	نحوه ♣ مصرف	مصرف روزانه (نخود)	روز در هفته	
تریاک	|__|	|__||__|	|__||__|	|__|	|__||__|	|__|	
هروئین	|__|	|__||__|	|__||__|	|__|	|__||__|	|__|	
سوخته	|__|	|__||__|	|__||__|	|__|	|__||__|	|__|	
شیره	|__|	|__||__|	|__||__|	|__|	|__||__|	|__|	

♣نحوه مصرف: 1= وافور، 2= خوراکی، 3= قورقوری، 4= سیمی، 5= تزریقی.

.9 مصرف الکل:

.9.1آیا بطور مرتب (حداقل یکبار در ماه به مدت شش ماه) از **مشروبات الکلی** استفاده کرده‌اید؟ |__| 1) بلی 2) خیر

.9.2 اگر بلی ، نوع و مدت مصرف آن را مشخص نمایید :

نوع	آیا مصرف شده؟ (1) بلی (2) خیر	اگر مصرف شده باشد				توضیحات
		از سن	تا سن	متوسط میزان مصرف (cc)در هر بار مصرف	تعداد دفعات	
آبجو	|__|					
مشروبات وارداتی (ودکا، ویسکی، جین،	|__|					
مشروبات دست‌ساز	|__|					درصد الکل دست‌ساز مصرفی ذکر شود
سایر	|__|					

.10فقط در مورد زنان:

.10.1 چند بار حامله شده‌اید؟..................

.10.2 چند فرزند زنده به دنیا آورده‌اید؟........................

.10.3 چند بار سقط کرده اند؟.....................

.10.4 سن شروع پریود؟ |__||__| (اگر نمی دا ند ، عدد 13 را وارد نمائید)

.10.5 سن شروع منوپوز؟ |__||__| (اگر هنوز منوپوز نشده، کد 98 و اگر نمی دا ند ، عدد 50 را وارد نمائید)

.10.6 سابقه نازایی : |__| 1) بلی 2) خیر

.10.7 سابقه مصرف داروهای ضدبارداری: |__| 1) بلی 2) خیر

10.7.1 . اگر بلی, برای چند سال؟ |__||__|

.24جمع آوری نمونه های بیولوژیک، الصاق پاسخهای CT و آندوسونوگرافی

.24.3 **نمونه خون** گرفته شد؟ |__| 1) بلی 2) خیر

24.4 کپی آزمایشهای خون : |__| 1) بلی 2) خیر

.24.5کپی گزارش CT: |__| 1) بلی 2) خیر

.24.6کپی گزارش سونوگرافی: |__| 1) بلی 2) خیر

.24.7کپی گزارش آندوسونوگرافی: |__| 1) بلی 2) خیر

.24.8 گپی گزارش ERCP : |__| 1) بلی 2) خیر

25 . آیا از ضایعه پانکراس یا مجاری صفراوی یا دوازدهه نمونه برداری شده است: |__| 1) بلی 2) خیر

.25.1 اگر نمونه برداری شده است به چه طریقی؟|__| **1**)آندوسونوگرافی 2)اندوسکوپی ERCP(3 4)جراحی 5)سایر (نام ببرید)...........

.25.2 اگر نمونه برداری شده از کجا انجام شده است؟

1)پانکراس |__| 2) مجاری صفراوی 3)دوازدهه 4) سایر(نام ببرید)................................

.25.3 محل بررسی نمونه : |__|

1)بیمارستان شریعتی 2) کلینیک مسعود 3)بیمارستان میلاد تهران

4)بیمارستان فیروزگر 5)بیمارستان اتیه 6) بیمارستان امام 7) سایر (نام ببرید)..............................

PBD|__|__|__|__|__|__| |__|__| |__|

28. لطفاً جدول زیر را کامل کنید ؟

<table>
<tr><td>تاریخ: شهر محل انجام: اسم مرکز انجام دهنده:
1- توده پانکراس : |__| 1) بلی 2) خیر
2- توده پری آمپولاری: |__| 1) بلی 2) خیر
3- اندازه CBD(mm): .... (ذکر نشده)
4- اندازه PD: :
4.1. در سر پانکراس(mm): ....
4.2. در تنه پانکراس (mm): ....
5- گرفتاری عروقی: |__| 1) بلی 2) خیر 3) ذکر نشده
5.1.1. اگر جواب بلی است کدام یک از عروق گرفتار است؟ |__|
1- PV 2-SMA 3-Aorta 4-SMV
6- LAP: |__| 1) بلی 2) خیر 3)ذکر نشده
6.1.1. اگر بلی کجا؟ |__|
1) ناف کبد 2) اطراف پورت 3)سلیاک 4)سایر
7- متاستاز کبدی: |__| 1) بلی 2) خیر 3)ذکر نشده
8- آسیت: |__| 1) بلی 2) خیر 3)ذکر نشده</td>
<td>28.1.CT شکم ؟ |__| 1
1) دارد 2)ندارد</td></tr>
<tr><td>تاریخ: شهر محل انجام: اسم مرکز انجام دهنده:
1-توده پانکراس : |__| 1) بلی 2) خیر
2-توده پری آمپولاری: |__| 1) بلی 2) خیر
3-اندازهCBD(mm) : ....
4-اندازه PD : 4.1.در سر پانکراس(mm): .... 4.2.در تنه پانکراس (mm): ....
5-گرفتاری عروقی: |__| 1) بلی 2) خیر 3)ذکر نشده
5.1.1. اگر جواب بلی است کدام یک از عروق گرفتار است؟ |__|
1- PV 2-SMA 3-Aorta 4-SMV
6- LAP: |__| 1) بلی 2) خیر 3)ذکر نشده
6.1.1. اگر بلی کجا؟ |__|
1)ناف کبد 2) اطراف پورت 3)سلیاک 4)سایر...............
7-متاستاز کبدی: |__| 1) بلی 2) خیر 3)ذکر نشده
8-آسیت: |__| 1) بلی 2) خیر 3)ذکر نشده
9- گسترش به دوازدهه: |__| 1) بلی 2) خیر 3)ذکر نشده
10-توده sub mucosal: |__| 1) بلی 2) خیر 3)ذکر نشده
10.1. اگر بلی توده زیر مخاطی کجا می باشد؟ |__| 1) مری 2) معده</td>
<td>28.2.آندوسونوگرافی |__|
1) دارد 2) ندارد</td></tr>
</table>

T= N= M= Stage=

PBD||__||__||__||__||__||__| |__||__| |__|

** (این قسمت توسط خانم دکتر پورشمس پر می شود)

26 .تشخیص: |__|

1) کلانژیوکارسینوما 2) آدنوکارسینومای پانکراس Peri-ampullary Ca.(3 4) کنترل

5)تومور اندوکرین پانکراس 6) سایر (نام ببرید)...............................

27.1. لطفاً جدول زیر را کامل نمائید (در مواردی که بیمار آن اطلاعات را ندارد کد 98 را وارد نمائید).

ردیف	آزمایش	نوبت اول		نوبت دوم		نوبت سوم	
		تاریخ:<br>شهر محل آزمایش:<br>اسم آزمایشگاه:		تاریخ:<br>شهر محل آزمایش:<br>اسم آزمایشگاه:		تاریخ:<br>شهر محل آزمایش:<br>اسم آزمایشگاه:	
1	Hb						
2	PT						
3	AST						
4	ALT						
5	Alk P						
6	γGT						
**7**	**بیلی روبین توتال**						
**8**	**بیلی روبین مستقیم**						
9	CA19.9						
10	CEA						
11	FBS						
12	HBs Ag	+1	–2	+1	–2	+1	–2
13	Anti HCV	+1	–2	+1	–2	+1	–2

30. گروه خونی : |__| A(1 B(2 AB(3 O(4 30.1.2 Rh+ 30.1.1 Rh-.

31.1.:جواب بیوپسی : |__|

1) الصاق شد 2) الصادق نشد 3) توضیح...

نام و نام خانوادگی پرسشگر

(این قسمت توسط دکتر پورشمس پر می شود)

31.2 .آیا آسیب شناسی دارد ؟ |__| 1) بلی 2) خیر

31.2.1 .اگر بلی است کدام یک از کدهای پاتولوژی می باشد؟ |__|

کدهای پاتولوژی:

1-inadequate sample 2-Adenocarcinoma 3-highly suspicious for malignancy
4-normal 5- pancreatitis 6-autoiune pancreatitis 7- lymphoma 8- Neuro-endocrine tumor
9-Cholangio-Carcinoma 10 -others …………….

PBD||__||__||__||__||__| |__||__| |__|

32.پیگیری ماه اول: |__| 1) دارد 2) ندارد **اگر دارد** :

32.1.1. **تاریخ**:........................

32.1.2.روش پیگیری: |__| 1)تلفن 2)ویزیت 3)سایر(نام ببرید): ....

32.1.3.وضعیت بیمار: |__| 1) alive 2) death تاریخ دقیق آن ذکر شود / /

32.1.4. اقدامات انجام شده: |__| و|__|

1.1	عمل جراحی درمانی	تاریخ عمل:	شهر محل عمل:	بیمارستان:
1.2	عمل جراحی کمکی	تاریخ عمل:	شهر محل عمل:	بیمارستان:
2	ERCP و گذاشتن Stent	تاریخ:	شهرمحل ERCP:	بیمارستان:
3	شیمی درمانی	تاریخ:	شهرمحل شیمی درمانی:	بیمارستان:
4	اقدام خاص انجام نشده است			
5	سایر (نام ببرید)			

نام و نام خانوادگی پرسشگر

33.پیگیری ماه دوم: |__| 1)دارد 2) ندارد **اگر دارد** :

33.1.1. **تاریخ**:........................

33.1.2.روش پیگیری: |__| 1)تلفن 2)ویزیت 3)سایر: نام ببرید..

33.1.3.وضعیت بیمار: |__| 1) alive 2) death تاریخ دقیق آن ذکر شود / / 33.1.4. اقدامات انجام شده :|__| و|__|

1.1	عمل جراحی درمانی	تاریخ عمل:	شهر محل عمل:	بیمارستان:
1.2	عمل جراحی کمکی	تاریخ عمل:	شهر محل عمل:	بیمارستان:
2	ERCP و گذاشتن Stent	تاریخ:	شهرمحل ERCP:	بیمارستان:
3	شیمی درمانی	تاریخ:	شهرمحل شیمی درمانی:	بیمارستان:
4	اقدام خاص انجام نشده است			
5	سایر (نام ببرید)			

نام و نام خانوادگی پرسشگر

34. پیگیری ماه سوم: |__| 1)دارد 2) ندارد **اگر دارد**:

34.1.1. **تاریخ**:........................

34.1.2.روش پیگیری: |__| 1)تلفن 2)ویزیت 3)سایر(نام ببرید) ....

34.1.3.وضعیت بیمار: |__| 1) alive 2) death تاریخ دقیق آن ذکر شود / / 4.1.4.اقدامات انجام شده :|__| و|__|

1.1	عمل جراحی درمانی	تاریخ عمل:	شهر محل عمل:	بیمارستان:
1.2	عمل جراحی کمکی	تاریخ عمل:	شهر محل عمل:	بیمارستان:
2	ERCP و گذاشتن Stent	تاریخ:	شهرمحل ERCP:	بیمارستان:
3	شیمی درمانی	تاریخ:	شهرمحل شیمی درمانی:	بیمارستان:
4	اقدام خاص انجام نشده است			
5	سایر (نام ببرید			

نام و نام خانوادگی پرسشگر
