# Supplementary material for: Survival features, prognostic factors, and determinants of diagnosis and treatment among Iranian patients with pancreatic cancer, a prospective study
Source: PLoS One. 2020 Dec 4;15(12):e0243511. doi: 10.1371/journal.pone.0243511 (PMC7717574; doi:10.1371/journal.pone.0243511)
Supplement: S2 Questionnaire — (PDF) [file pone.0243511.s005.pdf]

PBD||__||__||__||__||__| |__||__| |__|

1. 1.1**نام** : .... 1.1.2**نام خانوادگی** : ....

Name ……. Surname ……..

معاینه آنتروپومتریک (وزن و قد بدون کفش و لباس‌های سنگین اندازه‌گیری شود).

Anthropometric measurements

1.1.3 **قد به** cm :.................... 1.1.4 **وزن به** Kg :.....................

Height (cm) …….. Weight (kg) …….

1.2**محل انجام مصاحبه**: |__|

Place of performing the interview

1)بیمارستان شریعتی 2)مرکز Cohort گلستان 3) بیمارستان آتیه

4)کلینیک مسعود 5) بیمارستان فیروزگر 6) بیمارستان امام 7)سایر: ....

1.5 **تاریخ مصاحبه**: روز/ماه/سال ..../......../........

Date if the interview

1.6 **محل تولد**: استان................ شهرستان .... شهر .... روستا.................................

Place of birth

. مشخصات **محل سکونت فعلی** : **1**- شهر **2**-روستا ***چند سال است؟***

Place and duration of current residence place

استان:............... شهرستان:................ شهر:................. روستا:...............

خیابان:..................... کوچه:................. پلاک:.....................

تلفن ثابت منزل:.................. پیش شماره منزل :.................. تلفن همراه:...............................

مشخصات همراه بیمار یا یکی از اعضای خانواده یا دوستان و همکاران :

Contact details

نام : .... نام خانوادگی:... نسبت آشنایی:...

تلفن ثابت منزل:.................. پیش شماره منزل :................. تلفن همراه:...............................

1.7 **جنس** : |__| 1)مرد 2)زن

Gender (Male/Female)

1.8**تاریخ تولد**: روز/ماه / سال ..../......./.......

Date of birth

1.9**سن** (سال کامل) : ....

Age

1.10.1**نژاد پدر بیمار**: |__|

1)فارس 2)ترک 3)ترکمن 4)گیلانی 5)مازندرانی 6)کرد 7) لر 8)سایر(نام ببرید) :..............

Ethnicity of the father (Persian / Truk / Turkmen / Gilani / Mazenderani / Kurd / Lor / Others)

1.10.2**نژاد مادربیمار** : |__|

1)فارس 2)ترک 3)ترکمن 4)گیلانی 5)مازندرانی 6)کرد 7) لر 8)سایر(نام ببرید) :...........

Ethnicity of the mother (Persian / Truk / Turkmen / Gilani / Mazenderani / Kurd / Lor / Others)

1.11.1 **وضعیت تاهل** : |__|

1)مجرد 2)متاهل 3)همسر مرده، بیوه 4)مطلقه 5)سایر:.................

Marital status (single / married / widowed / divorced / others)

PBD||__||__||__||__||__| |__||__| |__|

1.11.2. سن بیمار در زمان اولین ازدواج: |__||__|

Age at the time of first marriage

1.12. **سطح تحصیلات بیمار:** |__|

1) بی سواد 2)مساوی یا کمتر از 5 سال 3)بین 6 – 8 سال 4) بین 9-12 سال 5) تحصیلات عالی

Education level (None / ≤ 5 years / 6-8 years / 9-12 years / University)

1.13**سطح تحصیل سرپرست خانواده:** |__|

1)بی سواد 2) مساوی یا کمتر از 5سال 3) بین 6-8 سال 4) بین 9 تا 12 سال 5) تحصیلات عالی

Education level of the head of the house (None / ≤ 5 years / 6-8 years / 9-12 years / University)

1.14.**هویت فرد پاسخگو** به سوالات : |__| و |__|

1) خود بیمار 2) همسر بیمار 3) خویشاوندان دیگر 4) سایر افراد:(نام ببرید) ....

Who is answering the questions (the patient / patient's partner / patient's other family members / others)

. علایم و نشانه های بیماری فعلی

Signs and the symptoms of the current illness

4.1. آیا شما تا به حال علایم زیر راداشته‌اید؟ اگر بلی، اولین بار کی دچار این علایم شدید؟ (**معیار طولانی ترین علامت است**)

Have you had the following symptoms? If yes, when did you first noticed the corresponding symptom?

4.2. زمان شروع علایم:|__|

1) کمتر از یک ماه قبل 2)بین 1 تا 3 ماه قبل

3) بین 3 تا 6 ماه قبل 4)بیش از 6 ماه قبل 5) هرگز

Time of the symptoms onset (less than one month / 1-3 months / 3-6 months / more than 6 months ago / never)

1)بلی 2) خیر<br>1. yes 2. no	علایم<br>Symptoms	شماره
|__|	زردی<br>Jaundice	4.1.1
|__|	خارش<br>Pruritus	4.1.2
|__|	تب<br>Fever	4.1.3
|__|	لرز<br>Shivering	4.1.4
|__|	یبوست<br>Constipation	4.1.5
|__|	درد شکم<br>Abdominal pain	4.1.6
|__|	تغییر رنگ ادرار<br>Urine discoloration	4.1.7
|__|	تغییر رنگ مدفوع<br>Stool discoloration	4.1.8

4.3 آیا **کاهش وزن** داشته‌اید؟ |__| 1) بلی 2) خیر

Have you lost weight? (yes / no)

4.3.1 اگر بلی ، چند کیلو گرم ؟...............(نا مشخص = 98) |__||__|

If yes, how many kilograms did you lose?

4.3.2 آیا این کاهش وزن ارادی بوده است ؟(یعنی با رژیم غذایی) |__|

1) بلی 2) خیر 8) کاهش وزن نداشته ام

Was this weight loss voluntary (e.g. following a dietary program)? (yes / no / I did not lose weight)

5. سابقه شخصی بیماری :

Past medical history

مدت بیماری (سال) **اگر خیر = کد 98**<br>Duration of the diseases (if no = 98)	1)بلی 2)خیر 3) نمی دانم<br>1. yes 2. no 3. I do not know	بیماری<br>Disease	شماره
|__||__|	|__|	سابقه بیماری دیابت<br>Diabetes mellitus	5.1.3
|__||__|	|__|	سابقه بیماری ایسکمیک قلبی (شامل سکته قلبی و نارسائی قلبی)<br>Cardiac diseases (including myocardial infarction, heart failure, etc)	5.1.6
|__||__|	|__|	سابقه سکته مغزی<br>Stroke	5.1.7
|__||__|	|__|	سابقه افزایش فشار خون<br>Hypertension	5.1.7
|__||__|	|__|	سابقه هیپرکلسترولمی<br>Hypercholesterolemia	5.1.16
|__||__|	|__|	سابقه هیپرتری گلیسیریدمی<br>Hypertriglyceridemia	5.1.17
|__||__|	|__|	سابقه عمل جراحی کیسه صفرا<br>Cholecystectomy	5.1.22

PBD||___||___||___||___||___| |___||___| |___|

6.1. آیا شما مبتلا به کانسری که توسط پزشک تشخیص داده شده باشد, بوده‌اید؟ |___| 1) بلی 2)خیر

Have you ever beed diagnosed with cancer (yes / no)

6.1.1 اگر بلی چه نوعی از سرطان؟ |___||___|

If yes, what cancer type?

6.1.2 اگر بلی،سن در زمان تشخیص؟ |___||___|

If yes, at what age you were first diagnosed with cancer?

6.1.3 اگر بلی، آیا شیمی درمانی شده است؟|___| 1) بلی 2)خیر

If yes, were you treated with chemotherapy?

6.1.4 اگر بلی، چند بار شیمی درمانی شده است؟|___| 1) بلی 2)خیر

If yes, how many chemotherapy cycles did you receive?

6.1.3 اگر بلی، تاریخ اولین شیمی درمانی؟ |___||___| /D |___||___| / M |___||___||___||___|

If yes, what was the date of your first chemotherapy treatment?

کد نوع سرطان: 1= دهان یا حلق، 2= مری، 3= معده، 4= روده بزرگ، 5= لوزالمعده، 6= کبد، 7= حنجره، 8= ریه، 9=پوست، 10= پستان، 11= رحم، 12= پروستات، 13= مثانه، 14= خون، 15= سایر، 16= کیسه صفرا, 17= مجاری صفراوی, 18= با منشا نامشخص.

Codes for different cancer types (1. Pharynx 2. Esophagus 3. Stomach 4. Colon 5. Pancreas 6. Liver 7. Larynx 8. Lung 9. Skin 10. Breast 11. Uterine 12. Prostate 13. Bladder 14. Blood 15. Others 16. Gall bladder 17. Bile ducts 18. Unknown origin)

6.3 **سابقه ابتلا به کانسر در اعضای فامیل**: |___| 1) بلی 2) خیر اگر بلی است، در جدول زیر فهرست نمایید:

Family history of cancer (yes / no). If yes, fill the following table:

نسبت فامیلی<br>Relative code	کد نوع سرطان<br>Cancer code	سن تشخیص<br>Age at diagnosis
|___||___|	|___||___|	|___||___|
|___||___|	|___||___|	|___||___|
|___||___|	|___||___|	|___||___|

کد نسبت فامیلی: (1) پدر، (2) مادر، (3) برادر تنی، (4) خواهر تنی، (5) برادر ناتنی، (6) خواهر ناتنی، (7) پسر، (8) دختر، (9) پدربزرگ یا مادربزرگ، (10) همسر، (11) سایر خویشاوندان خونی درجه دو (عمو، عمه, دایی، خاله، عمو و عمه‌زاده، خاله و دایی‌زاده)

Relative code (1. Father 2. Mother 3. Brother 4. Sister 5. Half-brother 6. Half-sister 7. Son 8. Daughter 9. Grandfather / Grandmother) 10. Wife 11. Second degree relatives (uncle / aunt / cousin)

7- **سابقه مصرف دارو**:

Medication use history

7.1 آیا تاکنون دارویی را به صورت منظم مصرف نموده‌اید؟ |___| 1) بلی 2) خیر

Have you ever used a medication regularly?

7.2 اگر بلی، نام، مدت و دفعات مصرف را فهرست نمایید (کلیه داروها از جمله داروهای ضدبارداری داروهای تقویتی):

If yes, name the medication and indicate the duration and frequency of use:

	نام دارو<br>Medication name	مصرف فعلی<br>(1)بلی / (2)خیر<br>Current use<br>1. Yes 2. No	مدت (سال)<br>Duration (years)	دفعات مصرف<br>1) روزانه 2) هفتگی 3)ماهانه<br>Frequency of use (per day / week / month)	**دوز(میلی گرم)**<br>Dose (mg)
7.2.1	آسپیرین<br>Aspirin	|___|	|___||___|	|___|	
7.2.2	استاتین‌ها *<br>Statins	|___|	|___||___|	|___|	
7.2.3	انسولین<br>Insulin	|___|	|___||___|	|___|	

PBD||__||__||__||__||__| |__||__| |__|

7.2.4	متفورمین<br>Metformin	|__|	|__||__|	|__|	
7.2.5	گلی بن کلامید<br>Glibenclamide	|__|	|__||__|	|__|	

* منظور از استاتین ها داروهای لووستاتین ،آتروروستاتین ، سیموستاتین می باشد.

* Statins mean medications as Lovastatin, Atorvastatin, and Simvastatin

8.1 آیا بطور مرتب (حداقل یکبار درهفته به مدت شش ماه) **سیگار** کشیده‌اید؟ |__| (1) بلی (2) خیر

Have you ever smoked cigarettes regularly (at least weekly over a 6 month period)? (yes / no)

8.2. آیا در حال حاضر سیگار می‌کشید؟ |__| (1) بلی (2) خیر (3) هیچگاه سیگار مصرف نکرده‌ام

Do you still smoke cigarettes now? (yes / no / never smoked)

8.3. مصرف سیگار را از زمان آغاز مصرف منظم آن فهرست نمایید:

List cigarette consumption beginning with when you started to smoke regularly:

از سن<br>From age	تا سن<br>To age	نوع •<br>Type *	تعداد در روز<br>Cigarettes per day	روز در هفته<br>Days per week
		|__|	|__||__|	|__|
		|__|	|__||__|	|__|
		|__|	|__||__|	|__|
		|__|	|__||__|	|__|
		|__|	|__||__|	|__|

• **نوع**: 1- سیگار فیلتردار کارخانه‌ای 2- سیگار بدون فیلتر کارخانه‌ای 3- دستساز

* Type: 1=Factory-made Filtered; 2=Factory-made non filtered; 3=Hand-made

8.4. آیا بطور مرتب (حداقل یکبار در هفته به مدت شش‌ماه) **ناس**، **قلیان**، **چپق یا پیپ** استفاده کرده‌اید؟|__| 1)بلی 2) خیر

Have you ever used Nass, Hookah, Calumet, or Pipe regularly (at least weekly over a 6 month period)?

در صورت بلی، نوع و مدت مصرف آن را مشخص نمایید:

If yes, list consumption:

	نوع<br>Type	آیا مصرف شده؟<br>بلی (1)<br>Used?<br>Yes(1)	اگر مصرف شده باشد				توضیحات<br>Comment
			از سن<br>From age	تا سن<br>To age	دفعات معمول روزانه<br>Use per day	روز در هفته<br>Days per week	
8.4.1	ناس<br>Nass	|__|	|__||__|	|__||__|	|__||__|	|__|	
8.4.2	چپق<br>Calumet	|__|	|__||__|	|__||__|	|__||__|	|__|	
8.4.3	پیپ<br>Pipe	|__|	|__||__|	|__||__|	|__||__|	|__|	
8.4.4	قلیان<br>Hookah	|__|	|__||__|	|__||__|	|__||__|	|__|	

8.5. آیا بطور مرتب (حداقل یکبار در هفته در مدت شش ماه) **تریاک ـ موادمخدر** استفاده کرده‌اید؟ |__| (1) بلی (2) خیر

Have you ever used opium or other opiates like opium juice, heroin, or Sukhteh regularly? (at least weekly over a 6 month period)

8.5.1. اگر بلی ، نوع و مدت مصرف آن را مشخص نمایید:

If yes, please specify:

توضیحات<br>Comments	اگر مصرف شده باشد<br>If used					آیا مصرف شده؟<br>بلی (1)<br>Used? Yes(1)	نوع<br>Type
	روز در هفته<br>Days per week	مصرف روزانه (نخود)<br>Nokhods	♣نحوه مصرف<br>Route of administration	تا سن<br>To age	از سن<br>From age		
	|__|	|__||__|	|__|	|__||__|	|__||__|	|__|	تریاک<br>Opium
	|__|	|__||__|	|__|	|__||__|	|__||__|	|__|	هروئین<br>Heroin
	|__|	|__||__|	|__|	|__||__|	|__||__|	|__|	سوخته<br>Sukhteh
	|__|	|__||__|	|__|	|__||__|	|__||__|	|__|	شیره<br>Shireh

♣نحوه مصرف: 1= وافور، 2= خوراکی، 3= قورقوری، 4= سیمی، 5= تزریقی.

♣ Route of administration: 1= Vafoor; 2= Eating; 3= Ghoor-Ghoori; 4= Wire; 5= Injection; 6= Other.

9. مصرف الکل:

Alcohol consumption

9.1آیا بطور مرتب (حداقل یکبار در ماه به مدت شش ماه) از **مشروبات الکلی** استفاده کرده‌اید؟ |__| 1) بلی 2) خیر

Have you ever consumed alcoholic beverages regularly (at least once a week for at least 6 months)? (yes /no)

9.2 اگر بلی ، نوع و مدت مصرف آن را مشخص نمایید :

If yes, please specify:

توضیحات<br>Comments	اگر مصرف شده باشد				آیا مصرف شده؟<br>(1) بلی (2) خیر<br>Used? Yes(1)<br>No (2)	نوع<br>Type
	تعداد دفعات مصرف در ماه<br>Times per month	متوسط میزان مصرف در هر بار مصرف(cc)<br>Average use each time (cc)	تا سن<br>To age	از سن<br>From age		
					|__|	آبجو<br>Beer
					|__|	مشروبات وارداتی (ودکا، ویسکی، جین، غیره)<br>Imported Sprit (ex. Vodka, Whisky, Gin, etc.)
درصد الکل دستساز مصرفی ذکر شود<br>Specify alcohol %					|__|	مشروبات دستساز<br>Country spirit
					|__|	سایر<br>Others

PBD||__||__||__||__||__| |__||__| |__|

10.فقط در مورد زنان:

Only for women

10.1. چند بار حامله شده‌اید؟..................

How many pregnancies have you had?

10.2. چند فرزند زنده به دنیا آورده‌اید؟......................

How many births have you given?

10.3. چند بار سقط کرده اند؟....................

How many abortions have you had?

10.4. سن شروع پریود؟ |__||__| (اگر نمی دا ند ، عدد 13 را وارد نمائید)

Age at the first menstrual period?

10.5. سن شروع منوپوز؟ |__||__| (اگر هنوز منوپوز نشده، کد 98 و اگر نمی دا ند ، عدد 50 را وارد نمائید)

Age at the menopause?

10.6. سابقه نازایی : |__| 1) بلی 2) خیر

History of infertility? (yes / no)

10.7. سابقه مصرف داروهای ضدبارداری: |__| 1) بلی 2) خیر

History of using contraceptive pills? (yes / no)

10.7.1 . اگر بلی, برای چند سال؟ |__||__|

If yes, for how many years?

24.جمع آوری نمونه های بیولوژیک، الصاق پاسخهای CT و آندوسونوگرافی

Bio-samples collection, reports of the CT imaging, and endoscopic ultrasonography

24.3. **نمونه خون** گرفته شد؟ |__| 1) بلی 2) خیر

Blood sample withdrawn? (yes / no)

24.4 کپی آزمایشهای خون : |__| 1) بلی 2) خیر

Copy of blood tests? (yes / no)

24.5.کپی گزارش CT: |__| 1) بلی 2) خیر

Copy of CT imaging report? (yes / no)

24.6.کپی گزارش سونوگرافی: |__| 1) بلی 2) خیر

Copy of ultrasound report? (yes / no)

24.7.کپی گزارش آندوسونوگرافی: |__| 1) بلی 2) خیر

Copy of endoscopic ultrasonography report? (yes / no)

24.8. کپی گزارش ERCP : |__| 1) بلی 2) خیر

Copy of ERCP report? (yes / no)

25 . آیا از ضایعه پانکراس یا مجاری صفراوی یا دوازدهه نمونه برداری شده است: |__| 1) بلی 2) خیر

Any sample obtained from the pancreatic lesion / bile ducts / duodenum? (yes / no)

25.1. اگر نمونه برداری شده است به چه طریقی؟|__| **1**)آندوسونوگرافی 2)اندوسکوپی 3)ERCP 4)جراحی 5)سایر (نام ببرید)...........

If yes, how the sample was taken? (Endoscopic sonography / endoscopy / ERCP / Surgery / Others)

25.2. اگر نمونه برداری شده از کجا انجام شده است؟

1)پانکراس |__| 2) مجاری صفراوی 3)دوازدهه 4) سایر(نام ببرید)...............................

If a sample was obtained, where the sample was obtained from? (Pancreas / Bile ducts / Duodenum / Others)

25.3. محل بررسی نمونه : |__|

1)بیمارستان شریعتی 2) کلینیک مسعود 3)بیمارستان میلاد تهران

4)بیمارستان فیروزگر 5)بیمارستان اتیه 6) بیمارستان امام 7) سایر (نام ببرید)............................

Name of the medical center where the sample was taken

PBD||__||__||__||__||__| |__||__| |__|

28. لطفاً جدول زیر را کامل کنید ؟

Please complete the table below

<table>
<tr>
<td>تاریخ: شهر محل انجام: اسم مرکز انجام دهنده:
Date, name of the city and the medical center</td>
<td rowspan="2">28.1 CT شکم ؟ |__|
1) دارد 2)ندارد
Abdominal CT scan imaging
1. yes 2. no</td>
</tr>
<tr>
<td>1- توده پانکراس : |__| 1) بلی 2) خیر
Pancreatic mass (yes / no)
2- توده پری آمپولاری: |__| 1) بلی 2) خیر
Periampullary mass (yes / no)
3- اندازه CBD(mm): .... (ذکر نشده)
CBD size (mm)
4- اندازه :PD :
PD size
4.1 در سر پانکراس(mm): ....
At the head of pancreas (mm)
4.2 در تنه پانکراس (mm): ....
At the body of pancreas (mm)
5- گرفتاری عروقی: |__| 1) بلی 2) خیر 3) ذکر نشده
Vascular involvement (yes / no / not mentioned)
5.1.1 اگر جواب بلی است کدام یک از عروق گرفتار است؟ |__|
1- PV 2-SMA 3-Aorta 4-SMV
If yes, which of the vessels was involved?
6- LAP: |__| 1) بلی 2) خیر 3)ذکر نشده
Lymphadenopathy (yes / no / not mentioned)
6.1.1 اگر بلی کجا؟ |__|
1) ناف کبد 2) اطراف پورت 3)سلیاک 4)سایر
If yes, what lymph nodes? (hepatic hilar / periportal / celiac / others)
7- متاستاز کبدی: |__| 1) بلی 2) خیر 3)ذکر نشده
Liver metastasis? (yes / no / not mentioned)
8- آسیت: |__| 1) بلی 2) خیر 3)ذکر نشده
Ascites (yes / no / not mentioned)</td>
</tr>
<tr>
<td>تاریخ: شهر محل انجام: اسم مرکز انجام دهنده:
Date, name of the city and the medical center</td>
<td rowspan="2">28.2 آندوسونوگرافی |__|
1) دارد 2) ندارد
Endoscopic ultrasonography
1. yes 2. no</td>
</tr>
<tr>
<td>1- توده پانکراس : |__| 1) بلی 2) خیر
Pancreatic mass (yes / no)
2- توده پری آمپولاری: |__| 1) بلی 2) خیر
Periampullary mass (yes / no)
3- اندازه CBD(mm) : ....
CBD size (mm)
4- اندازه PD : 4.1 در سر پانکراس(mm): .... 4.2 در تنه پانکراس (mm): ....
PD size (at the head of pancreas (mm), at the body of pancreas (mm))
5- گرفتاری عروقی: |__| 1) بلی 2) خیر 3)ذکر نشده
Vascular involvement (yes / no / not mentioned)
5.1.1 اگر جواب بلی است کدام یک از عروق گرفتار است؟ |__|
1- PV 2-SMA 3-Aorta 4-SMV
If yes, which of the vessels was involved?
6- LAP: |__| 1) بلی 2) خیر 3)ذکر نشده
Lymphadenopathy (yes / no / not mentioned)</td>
</tr>
</table>

6.1.1. اگر بلی کجا؟ |__| 1)ناف کبد 2) اطراف پورت 3)سلیاک 4)سایر...............<br>If yes, which lymph nodes are involved? (hepatic hilar / periportal / celiac / others)<br>**7–متاستاز کبدی:** |__| 1) بلی 2) خیر 3)ذکر نشده<br>Liver metastasis? (yes / no / not mentioned)<br>**8–آسیت:** |__| 1) بلی 2) خیر 3)ذکر نشده<br>Ascites (yes / no / not mentioned)<br>9– گسترش به دوازدهه: |__| 1) بلی 2) خیر 3)ذکر نشده<br>Duodenal involvement (yes / no / not mentioned)<br>10–توده **sub mucosal**: |__| 1) بلی 2) خیر 3)ذکر نشده<br>Sub mucosal tumor (yes / no / not mentioned)<br>10.1. اگر بلی توده زیر مخاطی کجا می باشد؟ |__| 1) مری 2) معده<br>If yes, where is the sub mucosal tumor (1. Esophagus 2. Stomach)	

T= N= M= Stage=

26. تشخیص: |__|
1) کلانژیوکارسینوما 2) آدنوکارسینومای پانکراس 3).Peri-ampullary Ca 4) کنترل
5)تومور اندوکرین پانکراس 6) سایر (نام ببرید)................................
Final diagnosis (Cholangiocarcinoma / Pancreatic adenocarcinoma / Periampullary carcinoma / Control / Pancreatic endocrine tumors / Others)

27.1. لطفاً جدول زیر را کامل نمائید (در مواردی که بیمار آن اطلاعات را ندارد کد 98 را وارد نمائید).

ردیف	آزمایش<br>Lab test	نوبت اول<br>First time	نوبت دوم<br>Second time	نوبت سوم<br>Third time
		تاریخ:<br>شهر محل آزمایش:<br>اسم آزمایشگاه:<br>Date / city of the lab tests / name of the laboratory	تاریخ:<br>شهر محل آزمایش:<br>اسم آزمایشگاه:<br>Date / city of the lab tests / name of the laboratory	تاریخ:<br>شهر محل آزمایش:<br>اسم آزمایشگاه:<br>Date / city of the lab tests / name of the laboratory
1	Hb			
2	PT			
3	AST			
4	ALT			
5	Alk P			
6	γGT			
**7**	**بیلی روبین توتال**<br>Total bilirubin			
**8**	**بیلی روبین مستقیم**<br>Direct bilirubin			
9	CA19.9			
10	CEA			
11	FBS			
12	HBs Ag			
13	Anti HCV			

30. گروه خونی : |__| A(1 B(2 AB(3 O(4 30.1.2 Rh+ 30.1.1 Rh-.
Blood group

31.1:جواب بیوپسی : |__| 1) الصاق شد 2) الصادق نشد 3) توضیح.. (Biopsy report attached)

PBD||__||__||__||__||__| |__||__| |__|

31.2. آیا آسیب شناسی دارد ؟ |__| 1) بلی 2) خیر

Pathology report (yes / no)

31.2.1 اگر بلی است کدام یک از کدهای پاتولوژی می باشد؟ |__|

If yes, specify the histology?

کدهای پاتولوژی:

1-inadequate sample	2-Adenocarcinoma	3-highly suspicious for malignancy	
4-normal 5- pancreatitis	6-autoiune pancreatitis	7- lymphoma	8- Neuro-endocrine tumor
9-Cholangio-Carcinoma	10 -others …………….		

32.پیگیری: |__| 1) دارد 2) ندارد **اگر دارد :**

Follow-up questionnaire (yes / no)

32.1.1 **تاریخ:**......................

Date

32.1.2.روش پیگیری: |__| 1)تلفن 2)ویزیت 3)سایر(نام ببرید): ....

Method of follow-up (telephone / visit / others)

32.1.3.وضعیت بیمار: |__| 1) alive 2) death تاریخ دقیق آن ذکر شود / /

Vital status (alive / dead)
Date of the vital status report

32.1.4. اقدامات انجام شده: |__| و|__|

Therapeutics & procedures

1.1	عمل جراحی درمانی<br>Curative Surgery	تاریخ عمل: شهر محل عمل: بیمارستان:<br>Date of surgery / city of surgery / medical center name
1.2	عمل جراحی کمکی<br>Palliative surgery	تاریخ عمل: شهر محل عمل: بیمارستان:<br>Date of surgery / city of surgery / medical center name
2	ERCP و گذاشتن Stent<br>ERCP & Stent insertion	تاریخ: شهرمحل ERCP: بیمارستان:<br>Date / city of / medical center name
3	شیمی درمانی<br>Chemotherapy	تاریخ: شهرمحل شیمی درمانی: بیمارستان:<br>Date / city of / medical center name
4	اقدام خاص انجام نشده است	No specific treatment or procedure was performed
5	سایر (نام ببرید)	Others (please specify)
